# Supplementary material for: Coherent optomechanical state transfer between disparate mechanical resonators
Source: Nat Commun. 2017 Oct 10;8:824. doi: 10.1038/s41467-017-00968-9 (PMC5635001; doi:10.1038/s41467-017-00968-9)
Supplement: Supplementary file 1 — Supplementary Information [file 41467_2017_968_MOESM1_ESM.pdf]

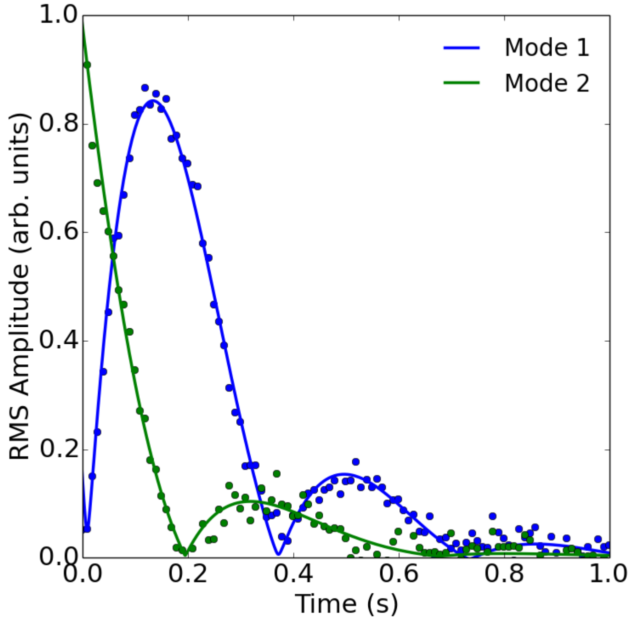

Supplementary Figure 1: Coherent optomechanical swapping between two membrane modes. The same experimental procedure from the main text is repeated with two mechanical modes of the resonator without the mirror. The system parameters for this plot are: $\omega_1/2\pi$ = 660 kHz, $\omega_2/2\pi$ = 1199 kHz and $\Delta/2\pi$ = 2.7 MHz. The solid lines are fits to the measured data points for each mode. Full coherent optomechanical swapping is also possible using only a membrane in the middle setup.

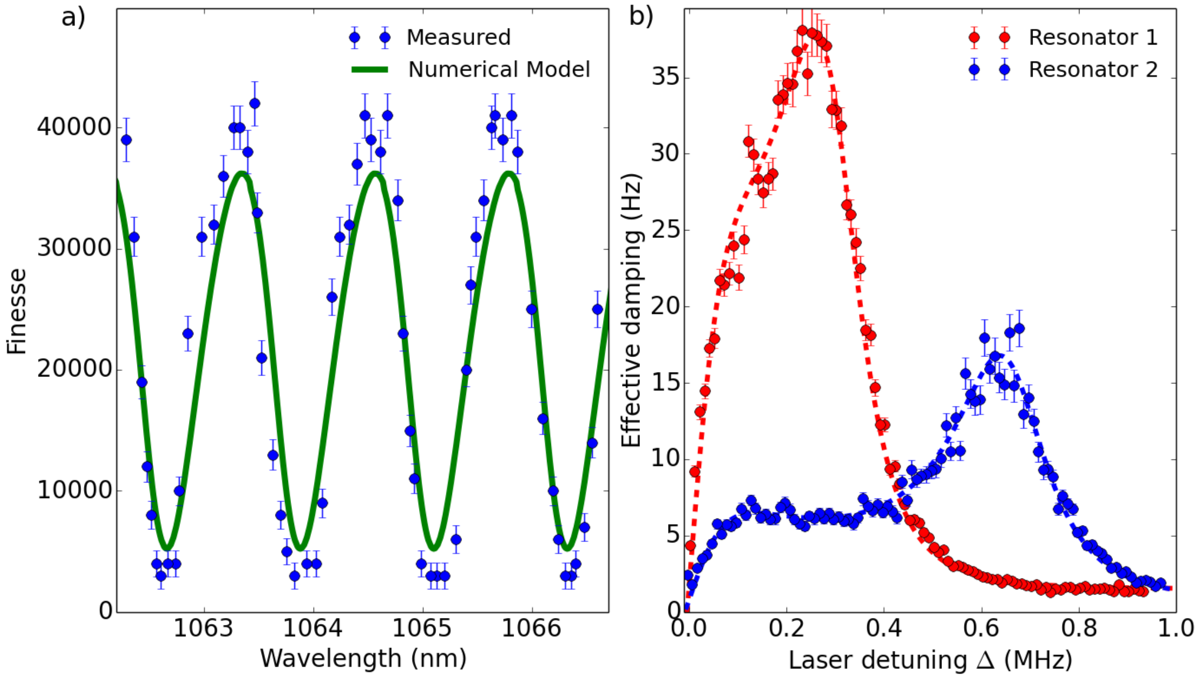

Supplementary Figure 2: Characterization of the hybrid membrane and moving end mirror cavity. a) Finesse is measured as a function of laser wavelength. Periodic variations in finesse are expected of a membrane in the middle system. The solid line is a numerical model using the transfer matrix method and two adjustable parameters, the imaginary index of the nitride film, n_{im}, and the thickness of the chip, t. **b)** We change the detuning of a single laser beam and measure the optical damping of each resonator independently. The dotted lines are fits to the theory of a single resonator, indicating that the hybrid system behaves as the sum of two linear optomechanical systems. Note that the separation of the two peaks shows that each resonator can be controlled independently. Error bars in **a)** reflect the standard deviation of statistical fluctuations between ten measurements and in **b)** indicate the deviations from a fit for the linewidth of each resonator.

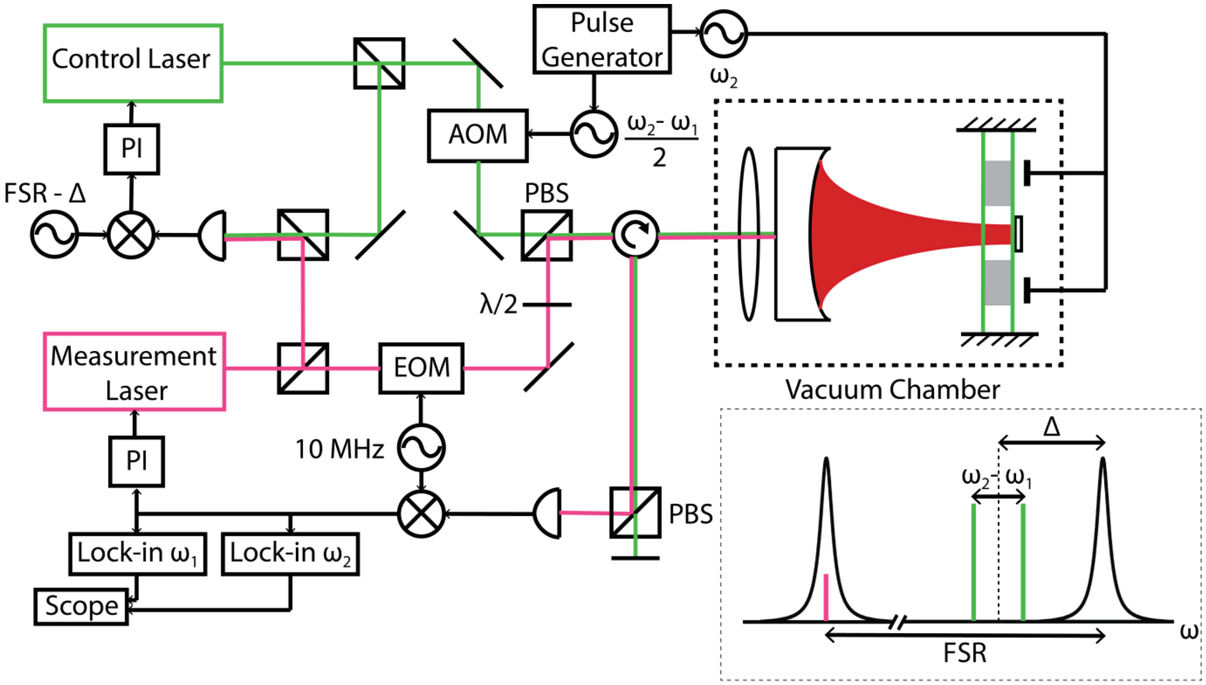

Supplementary Figure 3: Complete experimental setup. One measurement laser is locked to the optomechanical cavity, and used to read out the motion of the two resonators. A second control laser is locked to the first laser approximately one free spectral range (FSR) away, and the frequency separation is tuned to control the detuning, Δ. An acousto-optic modulator (AOM) generates the two laser tones at the mechanical difference frequency (ω_2-ω_1.) A pulse generator controls two function generators connected to the AOM and a ring electrode, which drives resonator 2 using the dielectric force. Other abbreviations are: electro-optic modulator (EOM), proportion integral feedback controller (PI) and polarizing beam splitter (PBS). The inset (bottom right) shows the frequencies of the measurement laser beam (pink) and control laser beam (green) input to the cavity relative to its optical resonances.

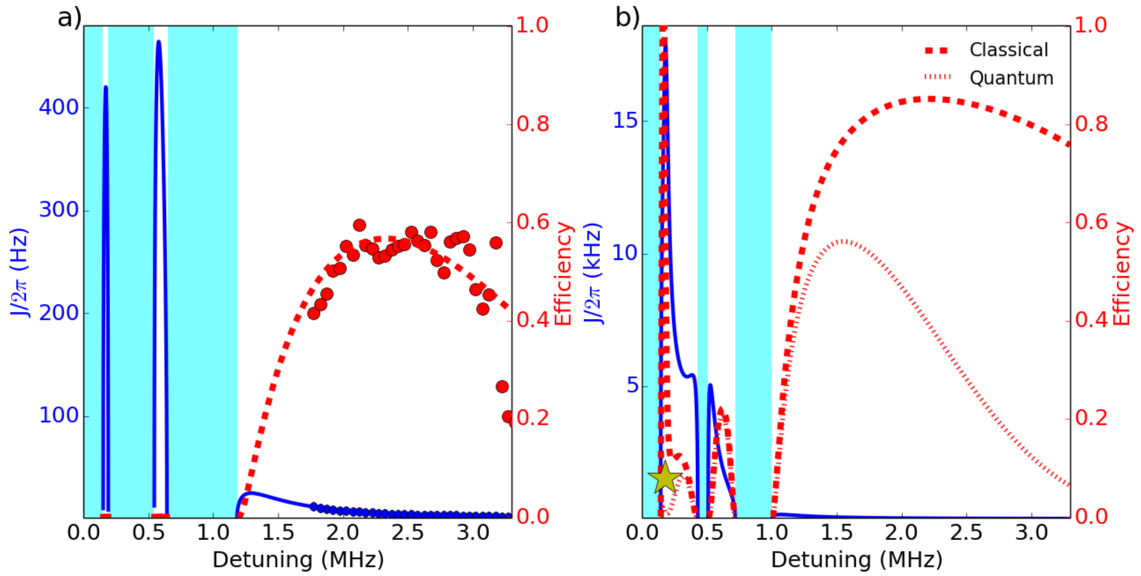

Supplementary Figure 4: Optomechanical swapping rate and efficiency. Theoretical predictions for optomechanical swapping rate, J, and state transfer efficiency of a π-pulse are shown for $\kappa/2\pi$ = 200 kHz and P_{in} = 65 μW in (**a**) and for $\kappa/2\pi$ = 50 kHz and P_{in} = 195 μW in (**b**). The shaded regions indicate detunings for which the coupled system is overdamped and full coherent state transfer is impossible. We note that by improving the finesse by a factor of 4, a point appears (at the star) where the classical losses go to zero. The maximum swap rate can be increased to 18 kHz and the state transfer efficiency to greater than 99% at this point. The quantum case is more limited, but can still reach reasonable efficiencies at the central detuning.
